# Identification of the Receptor and Cellular Ortholog of the Marek's Disease Virus (MDV) CXC Chemokine

**DOI:** 10.3389/fmicb.2017.02543

**Published:** 2017-12-15

**Authors:** Sonja Haertle, Ibrahim Alzuheir, Florian Busalt, Victoria Waters, Pete Kaiser, Benedikt B. Kaufer

**Affiliations:** ^1^Department für Veterinärwissenschaften, Ludwig-Maximilians-Universität München, München, Germany; ^2^Department of Veterinary Medicine, Institut für Virologie, Freie Universität Berlin, Berlin, Germany; ^3^Institute for Animal Health, Newbury, United Kingdom; ^4^Department of Avian Immunology and Pathology, The Roslin Institute and R(D)SVS, University of Edinburgh, Midlothian, United Kingdom

**Keywords:** MDV, chicken, vIL-8, CXCL13, CXCR5

## Abstract

Marek's disease virus (MDV) is a cell associated *alphaherpesvirus* that causes fatal lymphoma in chickens. One factor that plays a crucial role in MDV pathogenesis is the viral CXC chemokine vIL-8 that was originally named after chicken interleukin 8 (cIL-8). However, a recent study demonstrated that vIL-8 recruits B cells and a subset of T cells but not neutrophils, suggesting that vIL-8 is not a cIL-8 orthologue. In this study, we set to identify the cellular orthologues and receptor of vIL-8 using *in silico* analyses, binding and chemotaxis assays. Sequence and phylogenetic analyses of all chicken CXC chemokines present in the recently published chicken genome revealed that vIL-8 shares the highest amino acid similarity with the CXCL13L1 variant. To evaluate if vIL-8 and CXCL13L1 are also functional orthologues, we assessed their binding properties and chemotaxis activity. We demonstrated that both vIL-8 and CXCL13 variants bind B cells and subsets of T cells, confirming that they target the same cell types. In addition, the chemokines not only bound the target cells but also induced chemotaxis. Finally, we identified CXCR5 as the receptor of vIL-8 and CXCL13 variants and confirmed that the receptor is expressed on MDV target cells. Taken together, our data demonstrate the conservation of the receptor-ligand interaction between CXCR5 and CXCL13 and shed light on the origin and function of the MDV-encoded vIL-8 chemokine, which plays a crucial role in the pathogenesis of this highly oncogenic virus.

## Introduction

Marek's disease virus (MDV) is a highly oncogenic *Alphaherpesvirus* that infects chickens and causes immense economic losses worldwide (Davison and Nair, 2004). It is also known as gallid herpesvirus type 2 (GaHV-2) and causes a variety of clinical symptoms including immunosuppression, paralysis and acute death (Witter, 1997). In addition, MDV efficiently induces malignant T cell lymphomas, which are considered to be the most frequent clinically-diagnosed cancer in the animal kingdom (Parcells et al., 2012). Infection of susceptible animals with virulent MDV strains commonly results in a mortality of up to 100% (Davison and Nair, 2004). Current MDV vaccines are highly effective in minimizing commercial losses but do not elicit a sterilizing immunity, allowing a continued evolution of MDV strains in vaccinated chicken flocks (Davison and Nair, 2005; Osterrieder et al., 2006).

Upon inhalation of cell-free MDV from a contaminated environment, the virus is able to infect macrophages, dendritic cells (DCs) and B cells that transport the virus to lymphoid organs such as the spleen, thymus and bursa of Fabricius, where the virus can be detected within 24 h post-infection. In infected chickens, MDV predominantly replicates in B cells that subsequently transfer the virus to T cells (Jarosinski et al., 2006). The virus can either productively replicate in T cells or establish latency, allowing the virus to persist in the host for life (Jarosinski et al., 2006; Schermuly et al., 2015). In addition, MDV can transform CD4^+^ T cells, resulting in deadly lymphomas. MDV-induced tumor cells are often clonal within an animal and have a regulatory T cell (Treg) phenotype based on their cytokine and cell surface marker profiles including CD25 and CD30 (Burgess et al., 2004; Shack et al., 2008). In addition, these transformed cells harbor the integrated viral genome in one or multiple chromosomes (Kaufer et al., 2011b; Greco et al., 2014; Osterrieder et al., 2014), which ensures maintenance of the virus genome in these rapidly dividing cells. Several viral factors have been shown to be involved in the transformation process, including the major oncogene Meq (Jones et al., 1992), the virus-encoded telomerase RNA vTR (Kaufer et al., 2010, 2011a), miRNAs (Yao et al., 2009; Zhao et al., 2011) and several putative ORFs of unknown function (Jarosinski et al., 2005; Jarosinski and Schat, 2006).

Another factor that plays a crucial role in MDV pathogenesis and tumor formation is the CXC chemokine vIL-8 (Parcells et al., 2001; Engel et al., 2012). The MDV genome encodes two copies of vIL-8 that was originally named after interleukin 8 (cIL-8; cCXCL8), the first CXC chemokine identified in chicken (Kaiser et al., 1999; Parcells et al., 2001). vIL-8 is secreted by MDV infected cells and is essential for the establishment of infection in animals infected via the natural route. The viral chemokine was initially described as a chemoattractant of chicken peripheral blood mononuclear cells (PBMCs) (Parcells et al., 2001). Recent studies revealed that vIL-8 recruits B cells, the main target for MDV lytic replication. In addition, vIL-8 interacts with CD4^+^ CD25^+^ T cells that likely serve as a target for MDV latency and transformation (Engel et al., 2012).

The biological properties of vIL-8 are in stark contrast to the functions of its putative cellular IL-8 orthologues. IL-8 binds and recruits neutrophils instead of B or T cells (Kaiser et al., 2005), suggesting that IL-8 is not the cellular orthologue of the viral chemokine. Since the release of the complete chicken genome, eight chicken CXC chemokine orthologues have been identified (Kaiser et al., 2005). Amongst are the three inflammatory chemokines CXCL8L1 (K60), CXCL8L2 (9E3/CEF4) and CXCL1 (GROa) that possess a conserved ELR (glutamic acid–leucine–arginine) motif (Li et al., 2005; Poh et al., 2008). The other five are homeostatic ELR-negative CXC chemokines that coordinate leukocyte trafficking throughout the body. These include CXCL12, CXCL14 and three related genes named CXCL13L1, L2, and L3 (Kaiser et al., 2005).

Here, we performed sequence and phylogenetic analysis to identify potential cellular orthologues of vIL-8. The functional properties of vIL-8 and its closest relatives were assessed using binding and chemotaxis assays. Beyond that, we identified the receptor of vIL-8 and its cellular orthologues and confirmed receptor expression on the natural target cells of vIL-8.

## Materials and methods

### Sequence and phylogenetic analyses

Amino acid sequences of MDV vIL-8 and all known chicken CXC chemokines were aligned using the ClustalW (Vector NTI 9.1, Invitrogen) and MEGA 6 software (Tamura et al., 2013). Phylogenetic analyses of vIL-8, chicken and human CXC chemokines were performed based on their amino acid sequences applying neighbor-joining (NJ), maximum-parsimony (MP), and maximum-likelihood (ML) methods using the MEGA 6 software (Tamura et al., 2013). Full length amino acid sequences of MDV vIL-8 (AAN60433.1), the known chicken CXC chemokines, cCXCL1 (XP_420608.1), cCXCL8L1 (NP_990349.1), cCXCL8L2 (NP_990829.1), cCXCL12 (NP_989841.1), CXCL13L1 (XP_004941081.1), CXCL13L2 (CCC15119.1), CXCL13L3 (CCC15120.1), cCXCL14 (NP_990043.1) and human CXC chemokine orthologues, hCXCL1 (AAH11976.1), hCXCL8 (NP_000575.1), hCXCL12 (AAH39893.1), hCXCL13 (AAH12589.1), hCXCL14 (XP_527018.2) were obtained from Genbank.

### Cells

Cells for flow cytometric analysis were obtained from M11 (B^x2x^/ B^x2x^) white leghorn chicken. Birds were housed under conventional conditions in groups of up to 10 birds. All animal experiments were approved by the appropriate governmental agencies (Regierung von Oberbayern, Az.: 55.2-1-54-2532.6-12-09). HEK293 and HEK293-T cells were grown in DMEM high glucose supplemented with 10% FBS and 1% penicillin/streptomycin (Biochrom, Germany), DT40 cells were grown in IMDM (Biochrom, Germany) with 10% FBS, 1% chicken serum (ThermoFisher Scientific, USA) and 1 mM ß-Mercaptoethanol at 37°C. Primary leukocytes were obtained by dissociation of the organs using a stainless steel sieve and density centrifugation on Biocoll (1,077 g/ml, Biochrom, Germany).

### Generation of recombinant chemokines

To generate recombinant proteins for binding and chemotaxis assays, expression plasmids were generated. Full-length coding sequences of MDV-vIL-8, CXCL13L1, L2, or L3 were cloned into a modified pCR3.1 expression vector containing a C-terminal huFc-tag. cDNA from RB-1B infected cells or spleen cells of a Rhode Island Red (RIR) chicken served as a template for cloning the viral and cellular genes, respectively. HEK293 cells were transfected with the expression plasmids constructs using polyethyleneimine (PEI) as described previously (Boussif et al., 1995) or X-tremeGENE® 9 DNA transfection reagent (Sigma-Aldrich) according to the manufacturer's protocol. To generate protein for chemotaxis assays, cells were cultured in serum free HEK293 A medium (Bio&SELL, Germany). Supernatants containing the secreted huFc tagged chemokines or huFc control protein were harvested after 24 h and subjected to further analysis.

### Confirmation of recombinant chemokines

Recombinant chemokines and huFc control were quantified by ELISA. Briefly, 96-well ELISA plates (MaxiSorp, Nunc, Wiesbaden, Germany) were coated overnight with 1 μg/ml of donkey anti-huIgG (Jackson ImmunoResearch Europe Ltd, UK) in carbonate buffer (pH 9.6) and blocked with 4% skim milk. Plates were then incubated with serial dilutions of supernatants containing huFC-tagged chemokines, followed by incubation with HRP coupled rabbit anti-huIgG (SouthernBiotech, USA) 1:4,000 in PBS-T and tetramethylbenzidine (TMB). To confirm the correct size of the recombinant chemokines and huFc control protein we performed SDS-PAGE and Western blotting using a goat anti-human Fc HRP-conjugated secondary Ab (Invitrogen).

### Generation of CXCR5 expressing cells and a monoclonal anti-CXCR5 antibody

To generate cell lines expressing CXCR5 with an N-terminal extracellular Flag-tag, we amplified the full-length receptor from cDNA of bursal cells and cloned it into the p3XFLAG-myc-CMV™-25 expression vector (Sigma, USA). The CXCR5 expression plasmid was transfected into HEK293 cells using X-tremeGENE® 9 DNA transfection reagent (Sigma-Aldrich) according to the manufacturer's protocol and stable clones were selected with 250 μg/ml of neomycin (Biochrom, Germany). Receptor expression was confirmed by flow cytometry using an Alexa647 conjugated mouse anti-Flag antibody (AbD Serotec, Germany).

CXCR5 expressing HEK293 cells were used for repeated intraperitoneal immunizations of a BALB/c mouse. Murine spleen cells were fused to SP2/0-Ag14 hybridoma cells. Specificity of resulting hybridomas was examined by flow cytometry using undiluted hybridoma supernatant and goat anti-mouse-IgG-FITC (Sigma-Aldrich, USA, 1:200) on primary spleen cells as well as HEK293-CXCR5 cells and untransfected HEK293. Clone 6A9 was selected, its isotype was determined as IgG1 and monoclonality was ensured by subcloning using the single-cell limited dilution method.

### Binding assays

Receptor binding of recombinant chemokines was examined by flow cytometry on the chicken B cell line DT40 cells, HEK293-CXCR5 cells and primary chicken leukocytes from different lymphoid organs. Cells were incubated on ice with chemokine containing supernatants for 30 min. Chemokine binding was detected using a mouse anti-huFC-Alexa647 secondary antibody. Subpopulations of primary leukocytes were assessed by staining with RPE conjugated anti-Bu1 (clone AV20, B cells), anti-CD4 (CT4), anti-CD8 (CT8) and anti-Kul01 (myeloid cells) (Southern Biotechnology, USA).

### Migration assays

Migration assays were performed at room temperature with endotoxin-free single-use material. DT40 cells were washed twice in warm RPMI (Biochrom, Germany) and seeded at a concentration of 10^5^ cells/ml in chemotaxis medium (RPMI containing 0.5% bovine serum albumin). Twenty-four-well-Transwell® Permeable Supports (Corning, New York, USA) with a pore size of 5 μm were coated with bovine fibronectin (10 μg/ml) (Life Technologies) dissolved in endotoxin-free distilled H_2_O (Sigma-Aldrich) and incubated for 1 h at 37°C and 5% CO_2_. Plates were air-dried at 37°C for 2 h. Different dilutions of serum free supernatant from chemokine-transfected HEK293-T cells were added to the bottom of the transwell inserts. Chemotaxis medium served as a control. 100 μl of DT40 cell suspension was added to the transwell inserts. After a migration time of 90 min, cells were taken from the lower chamber and transferred directly into FACS Trucount® tubes (Becton Dickinson) and the number of cells was determined by flow cytometry. The chemokinesis index was calculated by dividing the number of migrated cells in stimulated wells by the chemokinesis control.

### Flow cytometry

Flow cytometric analyses were performed with a BD FACSCanto II (Becton Dickinson, Heidelberg, Germany) using DIVA and FlowJo (Tree Star Inc., Oregon, USA) software. Plots were gated for viable cells upon doublet discrimination.

### Statistics

Statistical analyses were performed using GraphPad Prism. Binding assays data of were analyzed using paired Student's *t*-test and migration assays by One-way ANOVA. Results were considered significant when *p* < 0.05.

## Results

### Sequence and phylogenetic analyses of MDV vIL-8

**Figure 1:**
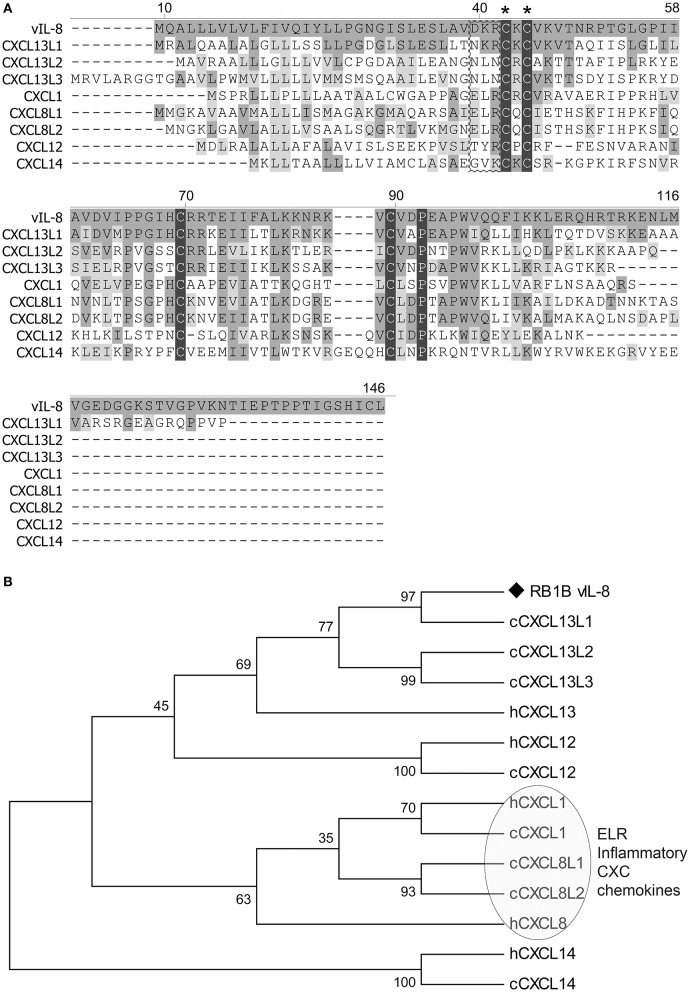
Sequence and phylogenetic analyses. **(A)** Amino acid sequence alignment of vIL-8 and chicken CXC chemokines was performed using Clustal W. The CXC and the ELR motif are indicated by asterisks and a box, respectively. Conserved cysteine and proline residues are highlighted with black shades. Conserved amino acid residues with vIL-8 are shown in dark gray and similar residues in light gray. Alignment gaps are indicated by dashes. **(B)** Phylogenetic tree of vIL-8, chicken (c) and related human (h) CXC chemokines. The human and chicken ELR-positive inflammatory CXC chemokines are indicated.

To identify potential cellular orthologs of the viral chemokine, we analyzed the amino acid sequences of all chicken CXC chemokines present in the recently published chicken genome (Figure 1A). Surprisingly, vIL-8 had the highest homology to the CXCL13 variants and not the two chicken IL8 chemokines CXCL8L1 (K60) and CXCL8L2 (9E3/CEF4) it was initially named after. CXCL13L1 shared the highest sequence identity of 53.8% with vIL-8, suggesting that this variant is the true orthologue of the viral chemokine. CXCL13L2 (33.0%) and CXCL13L3 (36.1%) also shared a higher sequence identity with vIL-8, compared to the IL-8 variants CXCL8L1 (28.8%) and CXCL8L2 (31.1%). Importantly, vIL-8 and all three CXCL13 variants lack the conserved ELR motif present in granulocyte attracting, inflammatory chemokines such as IL-8. Furthermore, vIL-8, CXCL13L1, and CXCL1L2 are the only genes with three exons, while all other chicken CXC chemokines have four exons (Wang et al., 2005). In addition, we performed phylogenetic analyses of vIL-8 and cellular CXC chemokines (Figure 1B), in which vIL-8 clustered with the human and the chicken CXCL13 variants and not the inflammatory IL-8 chemokines, again indicating that vIL-8 is a CXCL13 orthologue.

### vIL-8 is the functional ortholog of CXCL13L1

**Figure 2:**
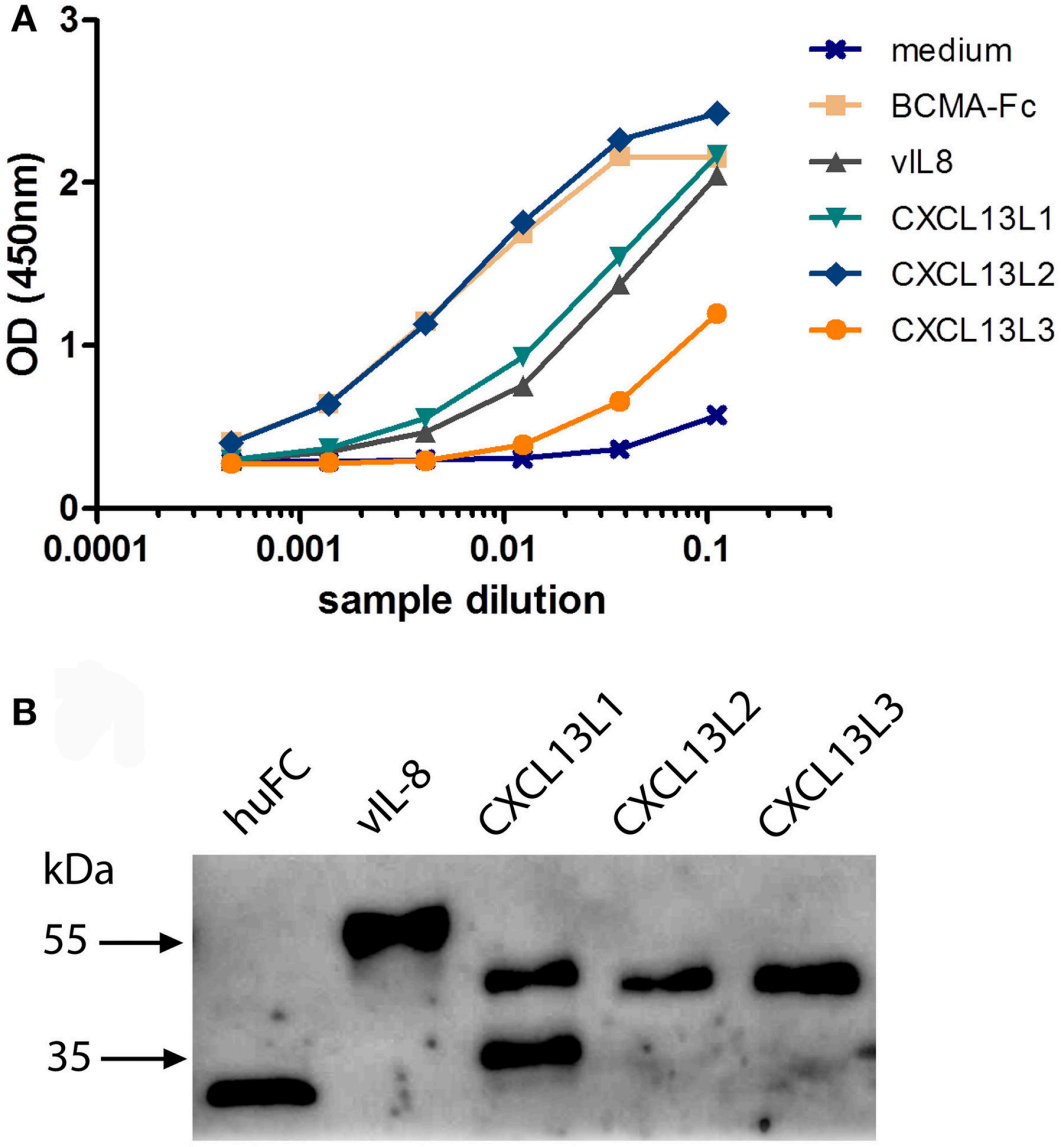
Expression of recombinant CXCL13 proteins. **(A)** Quantification of the indicated recombinant chemokines and the huFc control protein by ELISA. BCMA-Fc and media from mock transfected cells were used as positive and negative controls, respectively. **(B)** Western blot analysis of the indicated recombinant chemokines and the huFc control protein.

To confirm the *in silico* analysis, we compared the biological properties of vIL-8 and the chicken CXCL13 variants. To obtain functional chemokines, we cloned huFC-tagged vIL-8 and CXCL13 variants into expression plasmids and produced recombinant proteins in HEK293 cells. Recombinant protein in culture supernatants was quantified by ELISA (Figure 2A). Western blot analysis confirmed the correct size of the monomeric form of CXCL13L1, L2, and L3 of approximately 38 kDa (Figure 2B). For CXCL13L1, an additional lower molecular weight band was observed that most likely represents a degradation product of the chemokine. As observed previously, the size of huFC and vIL-8 was larger than predicted (Engel et al., 2012), which is likely due to glycosylation of the proteins.

**Figure 3:**
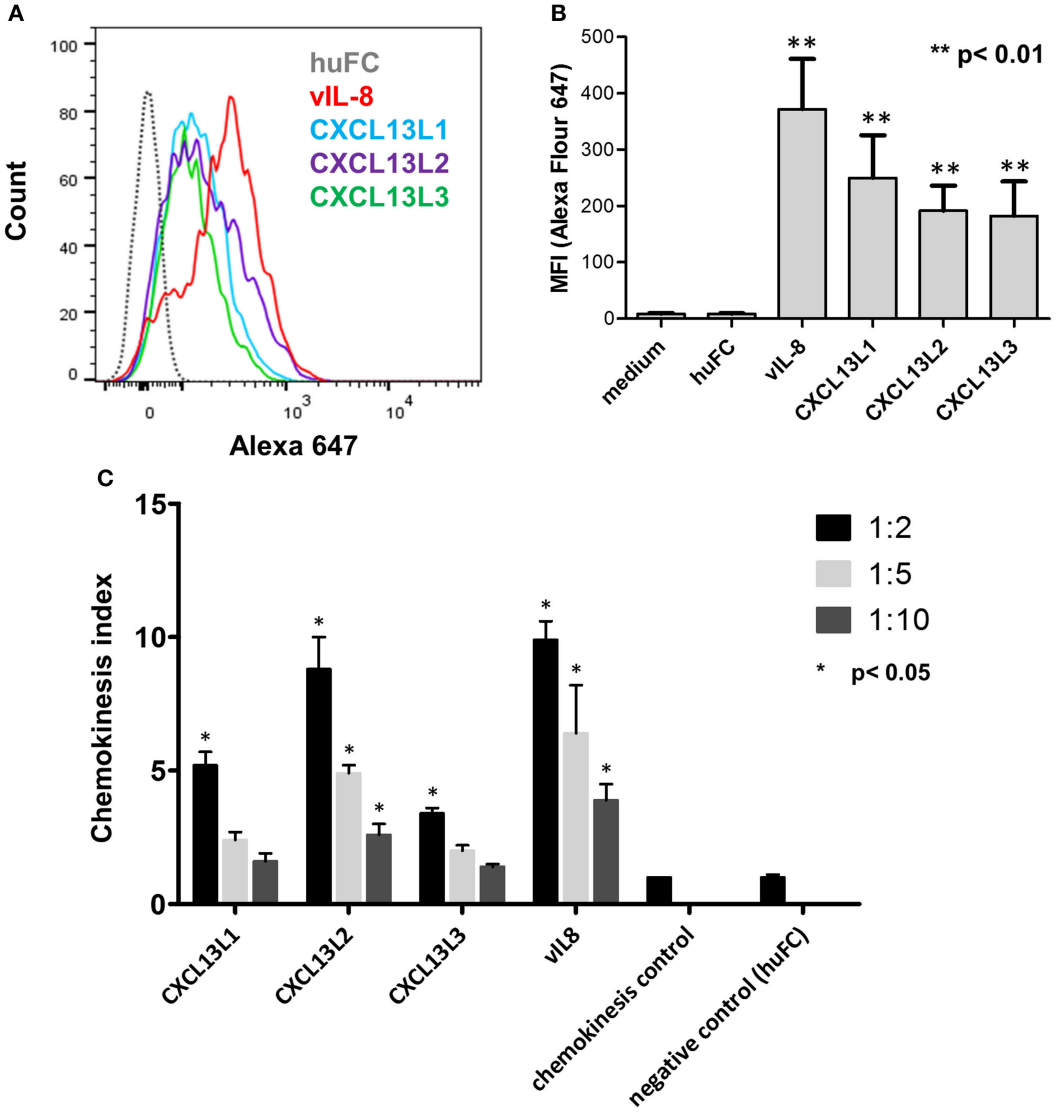
Functional analysis of vIL-8 and CXCL13 variants. **(A)** Binding of indicated chemokines to DT40 cells was assessed by FACS. One representative experiment of three independent experiments is shown. **(B)** Quantification of chemokine binding to DT40 cells shown in **(A)**. Mean ± SD of mean fluorescence intensity (MFI) of three independent experiments are shown. **(C)** Recruitment of DT40 cells was assessed by chemotaxis assays. Migration of B cells was assessed at three different dilutions as indicated. Shown are mean ± SD of four replicates out of two independent experiments.

We previously demonstrated that vIL-8 binds to and induces chemotaxis of B cells (Engel et al., 2012). Therefore, we assessed the binding of the recombinant chemokines to the chicken B cell line DT40. vIL-8 and all three CXCL13 variants efficiently bound to chicken B cells (Figures 3A,B), while no binding was observed for the control protein. Interestingly, vIL-8 showed a clearly higher MFI than the CXCL13 variants.

To determine if vIL-8 and the CXCL13 variants induce B cell migration, we performed chemotaxis assays using DT40 cells. vIL-8 and the CXCL13 variants efficiently induced chemotaxis (Figure 3C), suggesting that these chemokines have similar biological functions.

**Figure 4:**
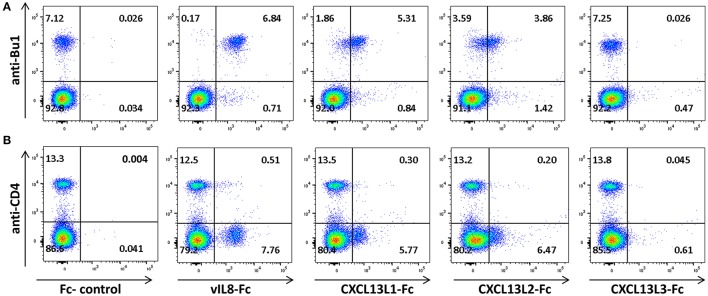
Interaction of vIL-8 and CXCL13 variants with primary lymphocytes. Binding of the indicated chemokines to **(A)** B and **(B)** T helper cells in primary PBMCs. Cells were stained with anti-Bu1 or anti-CD4 and the indicated chemokines and plots gated for leukocytes. Data represent one of two independent experiments.

To confirm that vIL-8 and the CXCL13 variants also target the same cell types *in vivo*, we performed binding assays with primary PBMCs. vIL-8, CXCL13L1 and CXCL13L2 bound to Bu1 positive B cells (Figure 4A), indicating that these chemokines also target the same cells *in vivo*. As on DT40 cells, strongest binding was observed for vIL-8. Surprisingly, CXCL13L3 did not bind primary B cells. In addition to B cells, vIL-8 was also previously shown to bind a certain subset of CD4^+^ T cells (Engel et al., 2012). Therefore, we assessed binding of vIL-8 and the CXCL13 variants to CD4^+^ T cells in primary PBMCs. vIL-8, CXCL13L1 and CXCL13L2 bound to a subpopulation of CD4^+^ T cells. As observed for B cells, vIL-8 bound these cells more efficiently than the cellular chemokines, while only minimal binding was observed for CXCL13L3 (Figure 4B). Taken together, our data demonstrate that vIL-8 has binding and chemotaxis properties comparable to CXCL13L1 and CXCL13L2, further supporting our *in silico* data that the viral chemokine is a CXCL13 ortholog.

### CXCR5 is a receptor of both vIL-8 and CXCL13

**Figure 5:**
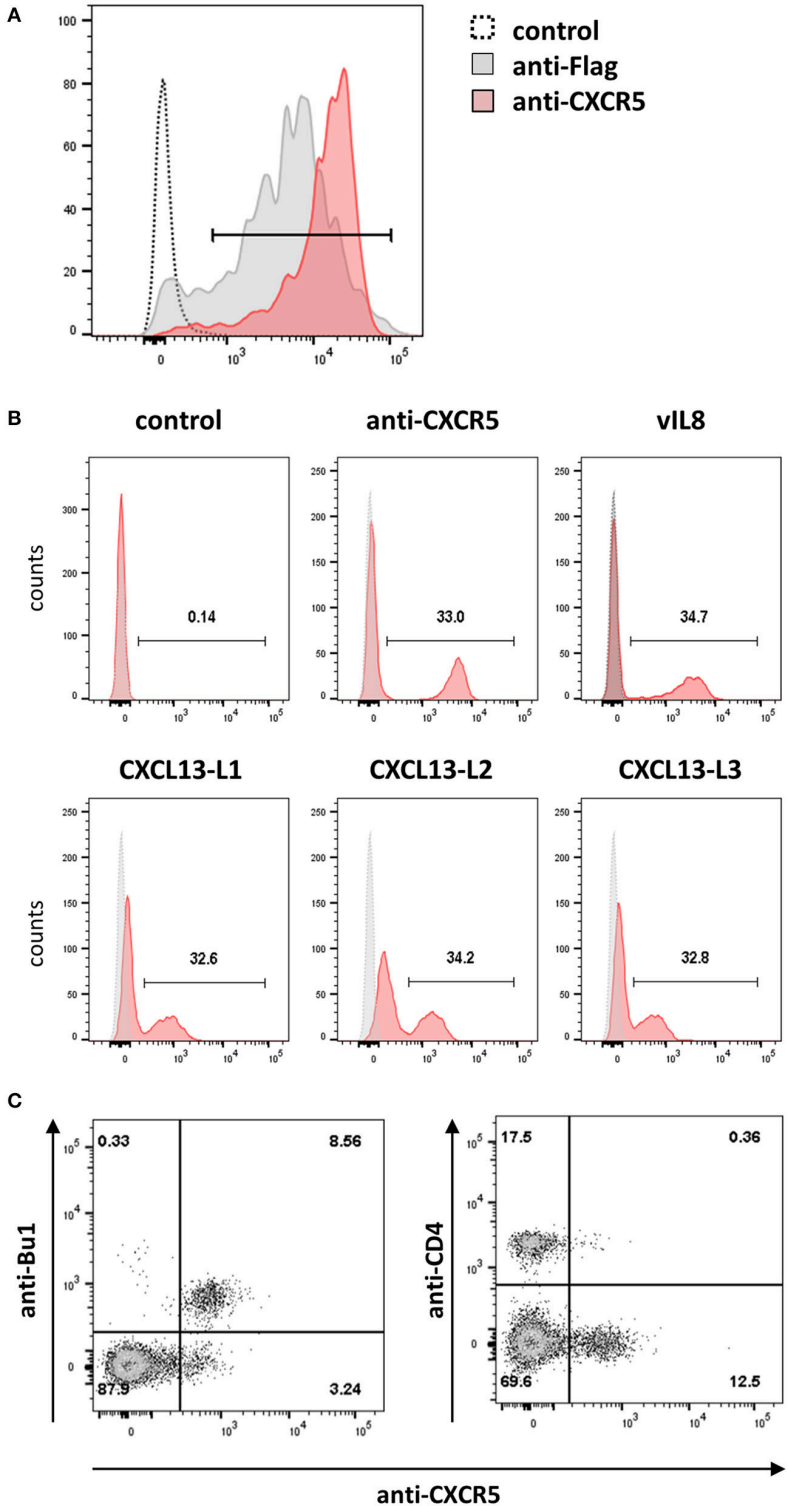
CXCR5 is the receptor for vIL-8 and CXCL13 variants. **(A)** The specificity of the newly generated mouse-anti-cCXR5 antibody (clone 6A9) was assessed by FACS on Flag-CXCR5 expressing and control HEK293 cells. **(B)** Binding of the indicated chemokines to a mixture of CXCR5 expressing HEK293 and untransfected parental cells (ratio 1:2). The huFc protein and CXCR5-specific antibody were used as negative and positive control, respectively. **(C)** Detection of the CXCR5 receptor on the target cells of vIL-8 and CXCL13 variants. Primary PBMCs were stained for B (Bu-1) or T helper cells (CD4) and the CXCR5 specific antibody.

Next, we set to determine the receptor of vIL-8 and its cellular orthologs. In humans and mice, CXCL13 has only a single receptor, CXCR5. Hence we examined whether the chicken CXCL13 orthologs bind to the recently discovered chicken CXCR5 (DeVries et al., 2006). Therefore, we generated HEK293 cells that express the receptor with an N-terminal Flag-tag and used these cells to produce a monoclonal antibody against chicken CXCR5, which stains HEK293-CXCR5 as well as an anti-Flag positive control (Figure 5A). To assess binding of vIL-8 and CXCL13 variants, CXCR5 expressing cells were mixed with parental cells at a 1:2 ratio as an internal control for binding specificity. As the anti-CXCR5 antibody, vIL-8 efficiently bound the CXCR5 expressing cells but not the control cells (Figure 5B), demonstrating that CXCR5 is the receptor of the viral chemokine. Similarly, all three CXCL13 variants interacted with CXCR5, highlighting that this receptor-ligand pair is conserved from mammals to chickens.

Next, we assessed if the CXCR5 is expressed on the natural target cells of MDV *in vivo*. We isolated primary cells from blood and different lymphoid tissues and detected CXCR5 expression using our CXCR5 antibody. CXCR5 was efficiently expressed on B cells and a subset of CD4^+^ cells as shown for vIL-8 (Figure 5C). In addition, we assessed CXCR5 expression in cells isolated from blood (Figure S1A), spleen (Figure S1B), bursa of Fabricius (Figure S1C) and thymus (Figure S1D). CXCR5 was found on all mature B cells in the spleen and all immature B cells in the Bursa of Fabricius. Very few CXCR5 positive T cells were observed in the blood, while about 10% of splenic CD4 and CD8 positive cells do express CXCR5. Among thymic T cells, CXCR5 expression is limited to small subpopulations of CD4 and CD8 single positive cells. Intriguingly, all KUL01^+^ monocytes in the blood and most of the KUL01^+^ macrophages in the spleen do also express CXCR5, suggesting the vIL-8 could also recruit these cells for MDV infection. Taken together, our data demonstrate that CXCR5 is the receptor of vIL-8 and CXCL13 and is expressed by B cells, CD4+ T cell subsets and monocytes /macrophages.

## Discussion

Herpesviruses co-evolved with their host over millions of years. During this time, many herpesviruses acquired host cell proteins that aid in the virus lifecycle by mediating immune evasion, cell proliferation or apoptosis control (Holzerlandt et al., 2002). In addition, several herpesvirus genomes encode viral chemokines that can induce or inhibit chemotaxis (Epperson et al., 2012; Cornaby et al., 2016). MDV acquired a CXC chemokine that was named vIL-8 due to its similarity to IL-8 (9E3/CEF4), the first CXC chemokine identified in chickens (Kaiser et al., 1999; Parcells et al., 2001). Since then, several other chicken CXC chemokines have been discovered in the complete chicken genome (International Chicken Genome Sequencing Consortium, 2004). Investigation of the biological functions of vIL-8 revealed that the virus chemokine binds and recruits different target cells than IL-8. Therefore, we set to identify the cellular ortholog of vIL-8 and compare their biological properties. *In silico* analyses provided the first evidence that vIL-8 is an ortholog of CXCL13, and not the inflammatory chicken IL-8 chemokines. The absence of the ELR motif and the exon structure also indicated that vIL-8 was derived from one of the CXCL13 variants, most likely CXCL13L1 and CXCL13L2. While CXCL13L1 shared the highest sequence similarity with vIL-8, the other two CXCL13 variants also possess similar biological properties. Binding and induction of chemotaxis of B cells (DT40) was comparable between vIL-8 and the CXCL13 variants. We used primary B and T cells in PBMCs to confirm that the binding also occurs to cells *ex vivo*. While CXCL13L1 and CXCL13L2 bound B and T cells in a manner comparable to vIL-8, CXCL13L3 showed no or only minimal binding. One possible reason for the reduced binding of the L3 variant is a lower stability of the chemokine. This could also explain the consistently lower yield of CXCL13L3 compared to the other chemokines as detected by ELISA. Alternatively, the amount of CXCR5 expressed on 293 cells vs. B and T cells could contribute to this phenomenon. Intriguingly, in both binding and migration assays, performance of vIL-8 seemed to exceed the cellular orthologs. Possibly, the viral chemokine has evolved an even higher affinity compared to its cellular orthologs to preferentially attract MDV target cells.

Only one CXCL13 chemokine is present in the genome of human and mice that binds to the CXCR5 receptor (Cyster et al., 2000). CXCR5 is mainly expressed on B cells, T helper cells and follicular helper T cells. Interaction between the homeostatic chemokine and its single receptor is central for the guidance of B cells to B cell follicles and has part in the zoning of Germinal Centers (Russo et al., 2014). It also attracts helper T cells to B cell areas and mediates the interaction between B and T cells.

In chickens, it remained unknown if the three CXCL13 variants also bind the putative orthologue of the CXCR5 receptor. Therefore, we generated a monoclonal antibody against CXCR5, addressed its expression on chicken lymphocytes and could demonstrate that all B cells and subsets of T cells have the receptor on their surface. Intriguingly these subsets are also targeted by vIL-8 and the CXCL13 variants. As vIL-8 and all CXCL13 variants efficiently bound to CXCR5 expressing cells, but not the control cells, we could confirm that chicken CXCR5 is the receptor for these chemokines. Recently it was shown, that macrophages are also a potential target for MDV infection (Chakraborty et al., 2017). Secretion of vIL-8 could allow the recruitment of CXCR5 expressing monocytes and macrophages to the site of infection that could transport the virus to lymphoid organs where B and T cells are infected.

Taken together, we analyzed vIL-8 and all annotated chicken CXC chemokines and identified the closest cellular orthologs. Our data demonstrates that vIL-8 has biological functions comparable to the CXCL13 variants and is most closely related to the CXCL13L1. In addition, we identified chicken CXCR5 as cellular receptor of this viral chemokine and its cellular orthologs. Our data thereby provide the first functional evidence that the chemokine-receptor pair CXCL13-CXCR5 is also conserved between mammals and an avian species. All in all, our data not only sheds light on the origin of the viral chemokine, but also provides important insights into the mechanism that allows vIL-8 to contribute to MDV pathogenesis.

## Author contributions

SH, IA, VW, PK and BK designed the study. SH, IA and FB performed experiments. SH, IA, FB and BK analyzed the data. SH, IA and BK wrote the manuscript.

### Conflict of interest statement

The authors declare that the research was conducted in the absence of any commercial or financial relationships that could be construed as a potential conflict of interest.

## Supplementary material

Figure S1 Leukocytes were isolated from blood **(A)**, spleen **(B)** and bursa of Fabricius **(C)**, stained with an anti-CXCR5 antibody and markers for different cell populations for flow cytometric analysis. **(D)** Cells from the thymus were subjected to a triple staining with anti-CD4, anti-CD8 and anti-CXCR5 and CD8 single positive (Q1), CD4/CD8 double positive (Q2), CD4 single positive (Q3) and double negative (Q4) cells were gated for CXCR5 expression.

## Abbreviations

MDV: Marek's disease virus
IL-8: interleukin 8
PBMCs: peripheral blood mononuclear cells.
