# Supplementary figures and images for: Identification of the Receptor and Cellular Ortholog of the Marek's Disease Virus (MDV) CXC Chemokine
Source: Front Microbiol. 2017 Dec 15;8:2543. doi: 10.3389/fmicb.2017.02543 (PMC5736565; doi:10.3389/fmicb.2017.02543)

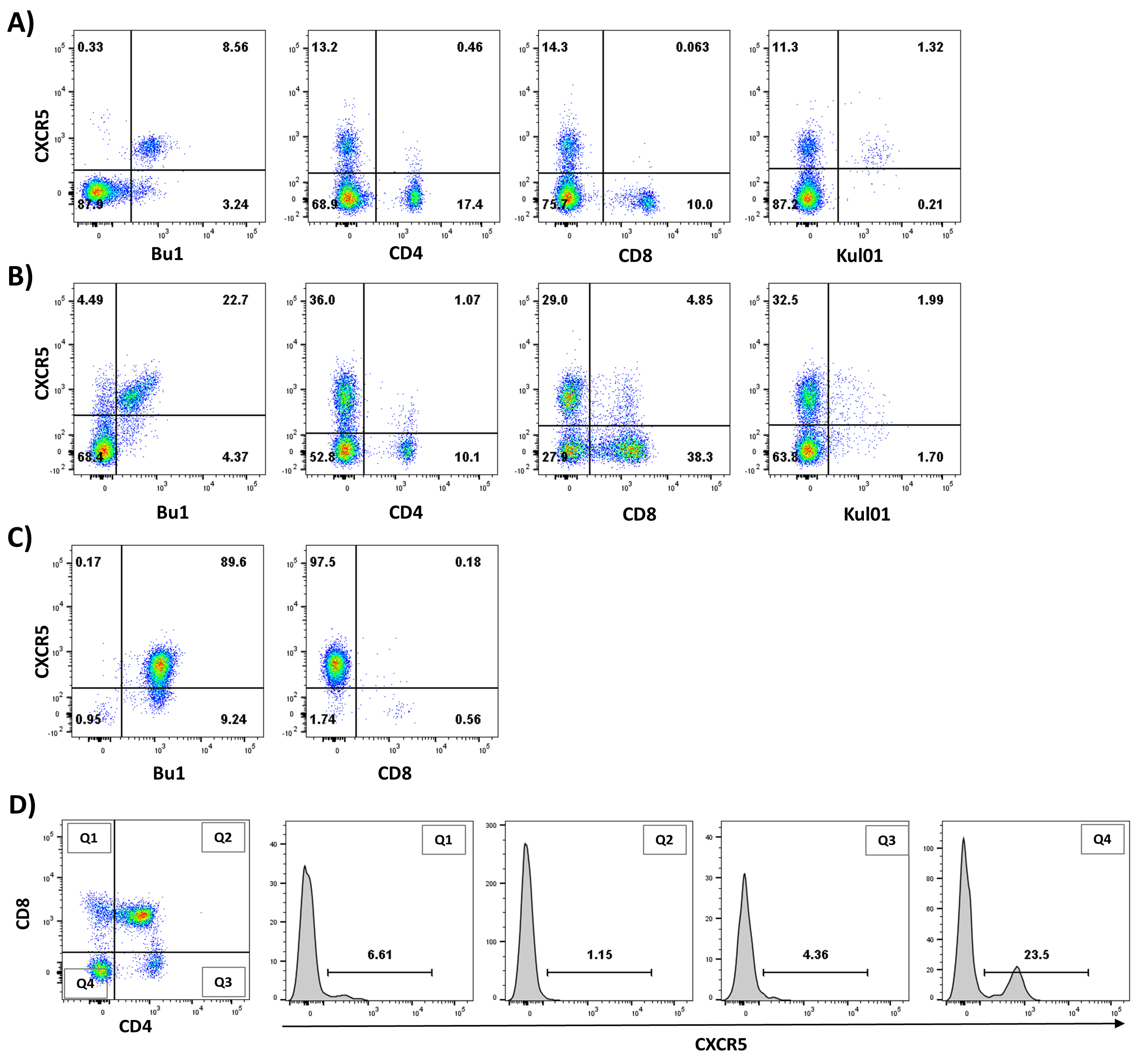

Supplement: Figure S1 — Leukocytes were isolated from blood (A), spleen (B) and bursa of Fabricius (C), stained with an anti-CXCR5 antibody and markers for different cell populations for flow cytometric analysis. (D) Cells from the thymus were subjected to a triple staining with anti-CD4, anti-CD8 and anti-CXCR5 and CD8 single positive (Q1), CD4/CD8 double positive (Q2), CD4 single positive (Q3) and double negative (Q4) cells were gated for CXCR5 expression. [file Image1.TIF]
